# An evolutionary perspective on the Crabtree effect

**DOI:** 10.3389/fmolb.2014.00017

**Published:** 2014-10-21

**Authors:** Thomas Pfeiffer, Annabel Morley

**Affiliations:** New Zealand Institute for Advanced Study, Massey UniversityAuckland, New Zealand

**Keywords:** yeast energy metabolism, Crabtree effect, evolution of metabolism, respiro-fermentation, evolutionary game theory

## Abstract

The capability to ferment sugars into ethanol is a key metabolic trait of yeasts. Crabtree-positive yeasts use fermentation even in the presence of oxygen, where they could, in principle, rely on the respiration pathway. This is surprising because fermentation has a much lower ATP yield than respiration (2 ATP vs. approximately 18 ATP per glucose). While genetic events in the evolution of the Crabtree effect have been identified, the selective advantages provided by this trait remain controversial. In this review we analyse explanations for the emergence of the Crabtree effect from an evolutionary and game-theoretical perspective. We argue that an increased rate of ATP production is likely the most important factor behind the emergence of the Crabtree effect.

## Introduction

**Figure 1:**
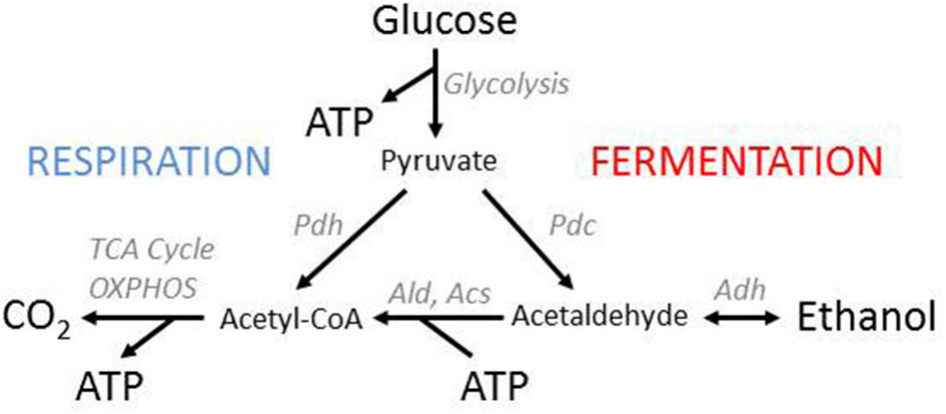
**Yeast energy metabolism**. Yeasts have two pathways for ATP production from glucose, respiration, and fermentation. Both pathways start with glycolysis, which results in the production of two molecules of pyruvate and ATP per glucose. In fermentation, pyruvate is then turned into ethanol. This process does not produce additional ATP but recycles the NAD^+^ consumed in glycolysis and thereby provides a way of oxygen-independent ATP production. In respiration, pyruvate is completely oxidized to CO_2_ through the TCA cycle and oxidative phosphorylation (OXPHOS), which yields additional ATP but requires oxygen. Crabtree positive yeasts, at sufficient levels of oxygen and glucose, use fermentation and respiration simultaneously. The ethanol that accumulates in the environment can be recycled for ATP production once glucose has been depleted. This process, however, yields less ATP than the direct oxidation of pyruvate because the synthesis of Acetyl-CoA from ethanol requires ATP.

Adenosine triphosphate (ATP) is a key compound in cellular energy metabolism, where it drives free-energy dependent processes such as motion, transport, biosynthesis and growth. Yeasts can typically use two different pathways to produce ATP from sugars, namely respiration and fermentation (Figure 1). While respiration results in a high yield of ATP (in *Saccharomyces cerevisiae* approximately 18 ATP per glucose), fermentation has a much lower ATP yield (2 ATP per glucose) but does not require oxygen. At high levels of sugar and oxygen, yeasts can produce ATP via respiration, fermentation, or a concurrent use of both pathways. Two strategies are commonly observed and relate to the well-known Crabtree effect (De Deken, 1966; Postma and Verduyn, 1989; Van Urk et al., 1990): the exclusive use of respiration in Crabtree-negative yeasts, and the simultaneous use of fermentation and respiration in Crabtree-positive yeasts. Crabtree-positive yeasts likely emerged around the same time as flowering plants, whose sugar-rich fruits and nectar might have provided a novel niche to ancestral yeast species (Piškur et al., 2006). A number of genetic events, such as a whole-genome duplication, regulatory rewiring of yeast energy metabolism and hexose transporter duplications, have likely contributed to the Crabtree effect (Wolfe and Shields, 1997; Piškur et al., 2006; Conant and Wolfe, 2007; Hagman et al., 2013). Yet, despite this mechanistic knowledge, the evolutionary forces behind the emergence of the Crabtree effect remain unclear. We here review explanations for the emergence of the Crabtree effect from an evolutionary and game-theoretical point of view. We start by giving an introduction of the metabolic system that underlies the respiro-fermentative metabolism of yeasts.

## Respiro-fermentation and the crabtree effect in yeasts

Sugars such as glucose are converted into pyruvate through glycolysis, a metabolic pathway employed with deviations in nearly all organisms (Berg et al., 2002). In the initial reactions of glycolysis, 2 ATP are consumed per glucose, and the resulting fructose 1,6-bisphosphate is transformed into two 3-carbon sugars. In the downstream reactions, the 3-carbon sugars are degraded to pyruvate, each yielding 2 ATP and 1 NADH.

Pyruvate can then be further degraded either through the respiration pathway or the fermentation pathway. In alcoholic fermentation, pyruvate is transformed into ethanol by pyruvate decarboxylase (Pdc) and alcohol dehydrogenase (Adh), resulting in a net gain of 2 ATP per glucose. Adh recycles the NADH that is formed in lower glycolysis back into NAD+ and thus alcoholic fermentation can operate in the absence of oxygen.

In the respiration pathway, pyruvate is transformed by pyruvate dehydrogenase (Pdh) into acetyl-Coenzyme A, which is then oxidized in the TCA cycle to CO_2_. This yields further GTP, NADH and other reduced co-enzymes which are then oxidized in the mitochondrion to produce ATP. Overall, the respiration pathway can in eukaryotes produce up to 38 molecules of ATP from one molecule of glucose, including the two produced by glycolysis. This value is substantially lower for organisms with a low P/O ratio in oxidative phosphorylation. For *S. cerevisiae*, where a P/O ratio of about 1.2 has been estimated, the resulting yield of respiration is about 18 ATP per glucose (Verduyn et al., 1991; van Gulik and Heijnen, 1995; de Kok et al., 2012).

While the capability of degrading sugars via fermentation allows yeasts to adapt to anoxic environments, the use of fermentation is not confined to such conditions. Some yeast species such as *S. cerevisiae* use fermentation even in the presence of oxygen, when glucose concentrations are sufficiently high. The use of fermentation in the presence of oxygen and at high glucose concentrations is referred to as the Crabtree effect (Crabtree, 1929). Yeasts that display a Crabtree effect are Crabtree-positive; yeasts that do not display a Crabtree effect are Crabtree-negative. Examples and growth data for Crabtree-positive and Crabtree-negative yeasts are given in Hagman et al. (2013).

The Crabtree effect can be easily demonstrated in chemostat (Postma and Verduyn, 1989) and batch culture (Verduyn et al., 1984). In glucose-limited chemostat culture and at low dilution rates, CO_2_ production equals O_2_ uptake. The biomass yield is high under such conditions (about 0.5 g/g) and the residual glucose concentration is low. As the dilution rate increases, the population is forced to replicate faster to maintain a steady state population size in the chemostat. This implicates an increase in the residual glucose concentration. Both CO_2_ production and O_2_ uptake increase with increasing dilution rate, but remain coupled until a critical point is reached. When the dilution rate is increased across this critical point, fermentation sets in. CO_2_ production increases rapidly and becomes uncoupled from O_2_ uptake. The onset of fermentation is accompanied by a sharp decline of biomass yield to values below 0.2 g/g (Postma and Verduyn, 1989).

The uptake of O_2_ remains constant (Postma and Verduyn, 1989) or even declines (Van Hoek et al., 1998). This is consistent with the view that respiration is confined to a maximal rate; if more sugar is available than can be processed through respiration, it is fermented. The decline in the rate of respiration, moreover, suggests that the limited capacity of respiration is not a “static” constraint. It likely reflects costs for the expression of enzymes, and the limited membrane space that is available to harbor the enzymes involved in oxidative phosphorylation. These factors might determine an upper limit for the flux through respiration, and favor a reduced rate of respiration once fermentation sets in (alternative and more detailed explanations are reviewed in Molenaar et al., 2009).

Batch culture experiments (Verduyn et al., 1984) allow for the establishment of the glucose concentration at which fermentation sets in. For *S. cerevisiae* this has been shown to happen at a glucose concentration of about 150 mg/l (for comparison, the uptake rate of glucose starts saturating around 500 mg/l), though this might vary from species to species and depend on the specific conditions. The sharp drop in biomass yield associated with fermentation raises the question of why Crabtree-positive yeasts use the “wasteful” fermentation pathway if they could in principle rely solely on respiration for ATP production.

## Evolution of the crabtree effect

A major event in the evolution of the *S. cerevisiae* lineage was a whole genome duplication (Wolfe and Shields, 1997; Kellis et al., 2004) that occurred approximately 100 million years ago (mya) and doubled the number of chromosomes from 8 to 16. The timing of the whole-genome duplication (WGD) coincides with the diversification of angiosperms (Wing and Boucher, 1998) which might have provided a novel niche for the ancestral yeasts. It has thus been argued that an increased flux in glycolysis has been an evolutionary beneficial outcome of the WGD (Liti and Louis, 2005; Merico et al., 2007). On the other hand, a comparative analysis of yeast species covering over 150 million years of yeast evolutionary history has also shown that while post-WGD lineages have a pronounced Crabtree effect, aerobic fermentation is not confined to these lineages (Merico et al., 2007). A number of additional evolutionary events have likely contributed in shaping the sugar metabolism of yeasts and are reviewed in Conant and Wolfe (2007); Merico et al. (2007); Hagman et al. (2013).

One event that has received particular attention is the duplication of alcohol dehydrogenase, giving rise to two distinctive enzymes, Adh1 and Adh2. This duplication event has led to one avenue of explanation for the use of “wasteful” fermentation by Crabtree-positive yeasts, a theory referred to as the “make-accumulate-consume” strategy (Piškur et al., 2006). The idea behind the “make-accumulate-consume” strategy (in short MAC) is that yeasts ferment glucose in order to defend sugar rich resources such as fruit from competitors by exploiting the toxicity of ethanol. Moreover, the ethanol can later be consumed, once the preferred carbohydrates have been depleted. Due to the resulting low ATP and biomass yield, fermentation in itself is seen as wasteful and “energetically expensive” (Thomson et al., 2005): even when no ethanol is lost to the environment and all of it can be recycled, there is a loss in terms of ATP due to the conversion of ethanol to acetyl-CoA by aldehyde dehydrogenase (Ald) and acetyl-CoA synthetase (Acs), which requires one additional ATP per ethanol (see Figure 1).

This explanation for the use of aerobic fermentation was specifically motivated by work resurrecting hypothetical common ancestors of Adh1 and Adh2 (Thomson et al., 2005). Enzyme kinetic parameters suggest that Adh 1 is more proficient in the excretion of ethanol, while Adh 2 is more proficient for the uptake of ethanol. The kinetics of the resurrected ancestral Adh has been shown to resemble the kinetics of Adh1 rather than Adh2. It has been argued that the role of ancestral Adh was to excrete ethanol under anaerobic conditions, rather than to consume it. Based on this it is stated that before the Adh duplication, ancestral yeast did not consume ethanol, and thus ancestral yeast did not accumulate ethanol under aerobic conditions; aerobic fermentation evolved after the Adh duplication.

An alternative view regarding the advantages and disadvantages of aerobic fermentation is based on trade-offs that emerge between rate and yield of ATP production, and consequently between growth rate and yield of an organism. This view has conceptually been outlined by Pfeiffer et al. (2001), and is referred to as the rate/yield trade-off hypothesis (in short RYT). While the ATP yield is the amount of ATP produced per unit of substrate, the rate of ATP production is the amount of ATP produced per unit of time. A trade-off between ATP rate and yield means that ATP can either be produced fast (i.e., at high rate and low yield) or efficiently (i.e., at low rate and high yield). Trade-offs between rate and yield of ATP production can emerge for various reasons, including fundamental thermodynamic constraints of ATP production (Pfeiffer and Bonhoeffer, 2002) and mechanistic constraints of a given energy metabolic pathway, such as costs imposed by the intermediates or enzymes of a pathway (Heinrich and Schuster, 1996; Pfeiffer and Bonhoeffer, 2004).

In the respiro-fermentative metabolism of yeasts, the relevant mechanistic constraint seems to be the limited capacity of the respiration pathway to produce ATP, which as discussed above, likely reflects costs and constraints for the expression of enzymes involved in respiration, and limited membrane space. When more sugar is available than can be processed through respiration, two strategies are feasible: (i) processing additional sugar through the fermentation pathway; or (ii) refraining from processing the additional sugar through the fermentation pathway. The first option is implemented by Crabtree-positive yeasts, the second by Crabtree-negative yeasts. Due to fermentation of additional glucose increasing the ATP production rate but lowering the overall ATP yield, the first option can be seen as a fast but inefficient strategy, while the second option can be seen as a slow but efficient strategy. According to RYT, for many micro-organisms under a wide range of environmental conditions, rate is more relevant for competitive fitness than yield. In other words, Crabtree-positive yeasts use aerobic fermentation because the increased rate of ATP production provides a selective advantage; the decreased yield is of little relevance for fitness.

The rate/yield trade-off hypothesis does not preclude that the toxicity of ethanol can contribute to the selective advantage of aerobic fermentation. However, MAC and RYT differ in one crucial point: whether or not fermentation provides a selective advantage through an increased rate of ATP production. If fermentation provides a selective advantage that is irrespective of the toxicity of ethanol, RYT provides a sufficient explanation for the emergence of the Crabtree effect. Anti-competitor effects of ethanol might have provided additional benefits, but these benefits are not necessary for explaining the emergence of the Crabtree effect.

As described above, evidence given in support for MAC comes from comparative analyses of yeast (Piškur et al., 2006; Merico et al., 2007; Rozpęedowska et al., 2011; Hagman et al., 2013), the study of the kinetic properties of ancestral Adh (Thomson et al., 2005) and the apparent disadvantage of a lower energetic efficiency of aerobic fermentation. Moreover, given that ethanol can kill microbial competitors it appears plausible that alcoholic fermentation emerged because it allows yeasts to defend resources. However, there is no direct support for the view that the selective advantage of aerobic fermentation relies on the toxicity of ethanol.

There are observations indicating that toxic effects on competitors are not a sufficient explanation for aerobic fermentation. Crabtree-positive yeasts are and have been kept in mono-culture—in the absence of competing microbial species—for industrial applications, in research labs and in controlled long-term evolution experiments (Ferea et al., 1999; Jasmin et al., 2012). To our knowledge, the loss of fermentation has not been observed under such conditions, even though this is expected if fermentation is energetically costly and the benefits arise solely from the toxic effects of ethanol on competitors. A loss of aerobic fermentation could, in principle, be easily achieved. Crabtree-negative variants of *S. cerevisiae* can be easily constructed by knocking out part of the repertoire of the yeast's hexose transporters (MacLean and Gudelj, 2006). Competition experiments between these constructs and the wild-type provide support for RYT.

An additional argument against MAC is that ethanol is already produced at comparably low concentrations of glucose (below 1 g/l), where ethanol cannot accumulate to toxic levels that are typically in a range of several grams per liter (Casey and Ingledew, 1986). Furthermore, shifts between energy metabolic pathways are not only observed in the respiro-fermentative metabolism of yeasts, but in a number of metabolic systems, including tumor cells (Warburg, 1956; Molenaar et al., 2009) and bacterial systems (Molenaar et al., 2009), and involve a range of end products that are not necessarily as toxic as ethanol. This highlights the need for a more universal explanation for metabolic shifts.

Direct experimental evidence for RYT in the context of yeast energy metabolism, however, has also remained elusive. An ideal approach would be to expose a *S. cerevisiae* population to selection for yield. Based on RYT, one would expect that fermentation is lost as the population evolves toward increasing yield and that fermentation is re-established after subsequent selection for rate. Unfortunately, such experiments have not been performed on yeast because experimental regimes that select for yield are much more difficult to implement than experimental regimes that select for rate. Recently however, a promising experimental setup for selection for yield has been developed based on inverse emulsions, and results for *Lactococcus lactis* support rate/yield trade-offs in this system (Bachmann et al., 2013). It remains to be tested whether similar results can be obtained for yeasts.

## Game-theoretical considerations

**Figure 2:**
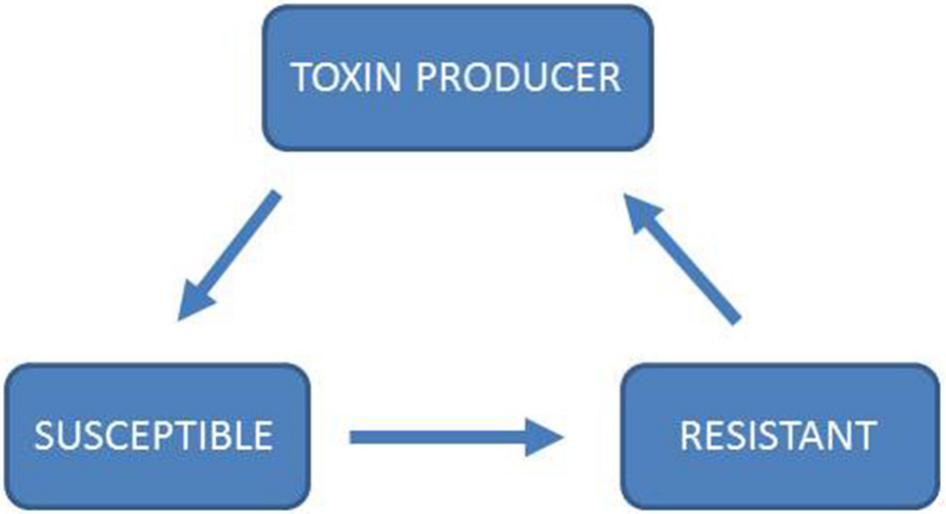
**Rock-Scissors-Paper dynamics of toxin production**. In the game Rock-Scissors-Paper, two players simultaneously have the choice between three strategies, “Rock,” “Scissors,” and “Paper.” Each strategy beats and is beaten by one other strategy: “Rock” beats “Scissors,” “Scissors” beats “Paper,” and “Paper” beats “Rock.” In this game there is no ESS as no one strategy can dominate both of the other strategies (Kerr et al., 2002; Nowak and Sigmund, 2004; Biernaskie et al., 2013). Rock-Paper-Scissor has been analyzed through models (Károlyi et al., 2005; Prado and Kerr, 2008; Biernaskie et al., 2013) and experimentally in plant systems (Lankau and Strauss, 2007; Cameron et al., 2009), bacterial systems (Kerr et al., 2002; Nahum et al., 2011) and lizards (Sinervo, 2001). In particular, toxin production in microbial systems has been shown to follow the rules of Rock-Paper-Scissor (Kerr et al., 2002; Nahum et al., 2011). In this system, toxin production is costly, as is resistance. A toxin-producing strain can out-compete a strain that is susceptible to the toxin. A resistant strain can out-compete the toxin-producing strain because it is resistant, but does not pay the costs of toxin production. In the absence of a toxin producer, the susceptible strain can out-compete the resistant strain because it does not pay the costs for resistance, thereby completing the cycle of mutual invasibility. If ethanol production and resistance to ethanol are costly traits, one might expect Rock-Paper-Scissor dynamics to influence the interactions between Crabtree-positive and Crabtree-negative yeasts.

Game theory is a mathematical tool to study interactions between “players” in strategic games (Von Neumann and Morgenstern, 1944). It has been shown to be a valuable tool for understanding social interactions between organisms, including micro-organisms (Nowak and Sigmund, 2004; Pfeiffer and Schuster, 2005; West et al., 2006). Rather than viewing evolutionary processes as fitness optimization that drives populations to move uphill in a static fitness landscape, game theory accounts for the fact that the players' strategies can change the fitness landscape. Thereby a dynamic landscape is created, where running uphill does not necessarily mean that one can ever arrive at a peak. In evolutionary game theory the notion of optimality is therefore replaced by evolutionary stable strategies (ESS) (Maynard Smith and Price, 1973). In a population where every player is playing the ESS strategy, any mutant with a different strategy has a fitness disadvantage and therefore cannot invade into the ESS population. An ESS is not guaranteed to exist for a given system, as is illustrated in the Rock-Scissors-Paper game (Figure 2). Applications of game theory to the evolution of biochemical systems have been previously reviewed (Pfeiffer and Schuster, 2005).

**Figure 3:**
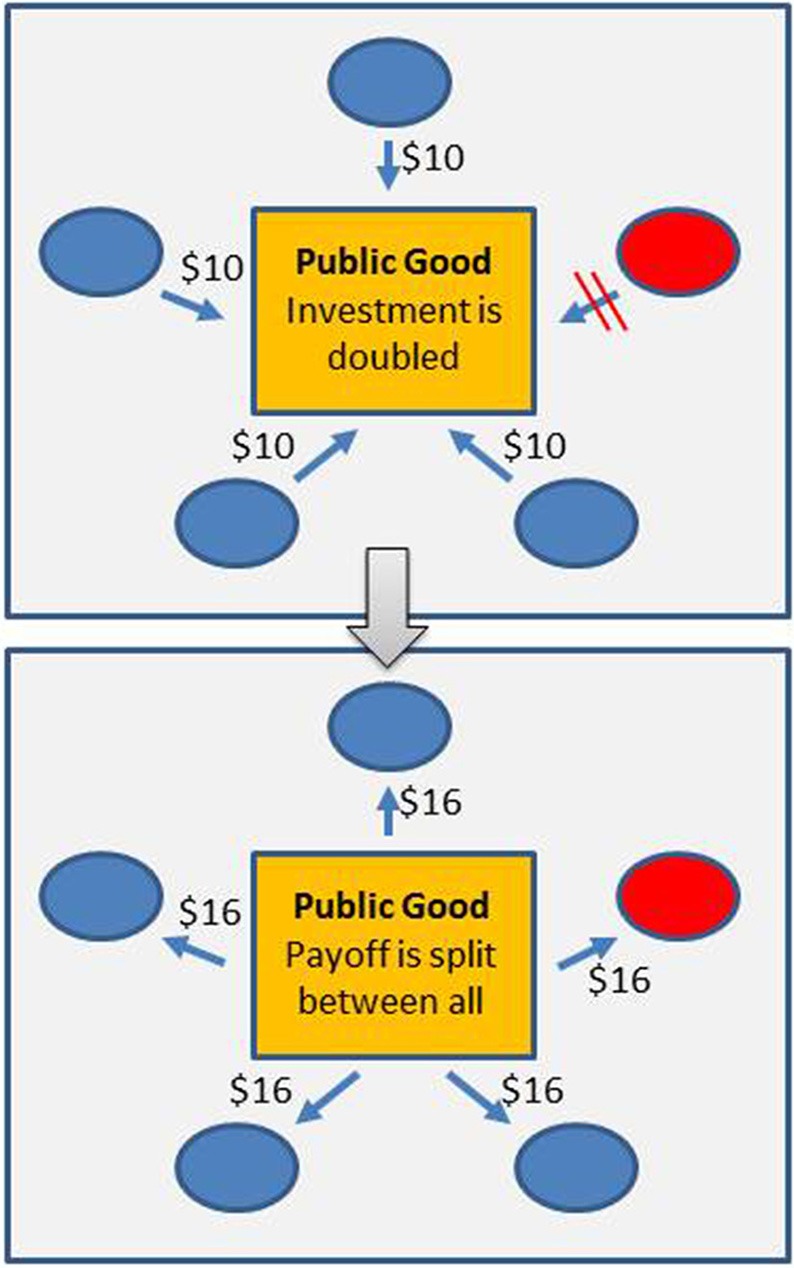
**Efficiency of resource use—a Public Goods Game**. In a Public Goods Game (or Tragedy of the Commons, Hardin, 1968), a number of players can invest in a public good. The returns from the investment are shared among all players, irrespective of the investment. Without any additional mechanisms in place, players that refrain from investing will receive a larger net payoff than players that do invest. In the figure, for instance, investments are doubled and then split evenly between all players. 4 players (shown in blue) invest $10, one does not invest (shown in red); everyone receives a return of $16. The net payoff for the investing players ($6 = $16 – $10) is smaller than the payoff for the player that does not invest ($16 = $16 – $0). The well-studied Prisoner's Dilemma (Rapoport, 1965) can be seen as a 2-player version of the Public Goods Game. For micro-organisms, a number of traits have been identified that can potentially create public goods, for example the excretion of exo-enzymes such as invertase (Greig and Travisano, 2004; West et al., 2006). Trade-offs between rate and efficiency in the use of shared resources have been shown to lead to a Public Goods Game. In the context of RYT this implies that aerobic fermentation can be seen as a selfish trait (Pfeiffer et al., 2001).

From a game theory perspective, MAC and RYT have very different characteristics. If aerobic fermentation is costly (excluding the beneficial effects from the toxicity of ethanol), the production of ethanol could be viewed as the creation of a public good; numerous similar instances of public good production by microbes have been identified in the past (West et al., 2006). This implies that a fermenting population would be prone to invasion of a “cheater” that benefits from the ethanol production of others but does not pay the costs of ethanol production. Ethanol production would not constitute an evolutionary stable strategy. Accounting for hypothetical differences in susceptibility/resistance to ethanol, MAC could perhaps lead to cycling of strategies as is observed for other anti-competitor toxin systems (see Figure 2). In contrast, RYT views aerobic fermentation as a strategy that destroys public goods due to its inefficiency: While efficient resource use is a trait that is of benefit at the group or population level, but not for the individual organism, inefficient resource use is of disadvantage for the group/population but of advantage for the individual (see Figure 3). It remains to be investigated if these differences can be exploited experimentally to determine whether MAC or RYT describes the costs and benefits of the Crabtree effect more appropriately.

## Metabolic fitness landscapes

Rate and yield, and the toxicity of end products are plausible but not necessarily the only fitness relevant properties of a metabolic pathway. Aside from ethanol, fermentation also leads to the production of heat, which in turn might have a detrimental effect on competitors (Goddard, 2008). Other potentially relevant factors may include costs paid for synthesizing the enzymes of a pathway, for the space that the enzymes take (especially for membrane-bound enzymes), for the osmotic constraints driven by the intermediates of a metabolic pathway (Heinrich and Schuster, 1996; Pfeiffer and Bonhoeffer, 2004; Molenaar et al., 2009; Zhuang et al., 2011; Goel et al., 2012), and for the effects of damaging side products such as reactive oxygen species (ROS) formed in respiration (Slavov et al., 2014). Moreover, a focus on the properties of ATP-producing (catabolic) pathways neglects the role of anabolism. With increasing rate of ATP production, ATP-consuming (anabolic) processes such as ribosome and protein synthesis might increasingly constrain growth (Scott et al., 2010; Kussell, 2013). Overall, metabolic systems, and shifts in the use of metabolic pathways, can be viewed to reflect a condition-dependent set of constraints in “cellular economics” (Molenaar et al., 2009). Within this context, RYT and MAC are relatively simplistic explanations that focus on (and disagree about) the most important single fitness-relevant factor behind the Crabtree effect.

Investigating the detailed costs and benefits associated with metabolic traits remains a promising field for future research, as are more comprehensive approaches to study the diversity of metabolic strategies used by microbes. In the context of yeasts, it will illuminate the origin of a fundamental trait in energy metabolism that is of substantial relevance for wine-making and industrial fermentation. In a more general context, such a research agenda is of importance for research fields ranging from metabolomics (e.g. for flux-distribution predictions) (Schuster et al., 2008; Molenaar et al., 2009) to the social evolutionary theory of micro-organisms (Pfeiffer et al., 2001; Pfeiffer and Bonhoeffer, 2004; Pfeiffer and Schuster, 2005; MacLean and Gudelj, 2006; Schuster et al., 2008). Moreover, it would generate a better understanding of metabolic strategies used by tumor cells (DeBerardinis et al., 2008; Diaz-Ruiz et al., 2011)—after all, tumor cell metabolism was one of the starting points leading to the discovery of the Crabtree effect almost a hundred years ago (Crabtree, 1929).

### Conflict of interest statement

The authors declare that the research was conducted in the absence of any commercial or financial relationships that could be construed as a potential conflict of interest.
